# Potential Developmental and Reproductive Impacts of Triclocarban: A Scoping Review

**DOI:** 10.1155/2017/9679738

**Published:** 2017-11-23

**Authors:** Johanna R. Rochester, Ashley L. Bolden, Katherine E. Pelch, Carol F. Kwiatkowski

**Affiliations:** ^1^The Endocrine Disruption Exchange (TEDX), Eckert, CO, USA; ^2^Department of Integrative Physiology, University of Colorado, Boulder, CO, USA

## Abstract

Triclocarban (TCC) is an antimicrobial agent used in personal care products. Although frequently studied with another antimicrobial, triclosan, it is not as well researched, and there are very few reviews of the biological activity of TCC. TCC has been shown to be a possible endocrine disruptor, acting by enhancing the activity of endogenous hormones. TCC has been banned in the US for certain applications; however, many human populations, in and outside the US, exhibit exposure to TCC. Because of the concern of the health effects of TCC, we conducted a scoping review in order to map the current body of literature on the endocrine, reproductive, and developmental effects of TCC. The aim of this scoping review was to identify possible endpoints for future systematic review and to make recommendations for future research. A search of the literature until August 2017 yielded 32 relevant studies in humans, rodents, fish, invertebrates, and* in vitro*. Based on the robustness of the literature in all three evidence streams (human, animal, and* in vitro*), we identified three endpoints for possible systematic review: estrogenic activity, androgenic activity, and offspring growth. In this review, we describe the body of evidence and make recommendations for future research.

## 1. Introduction

Triclocarban (TCC) is an antimicrobial agent primarily used in bar soaps, plastics (including toys, tableware, and utensils), cleansing lotions, and wipes [1, 2]. Although TCC and triclosan are frequently discussed and studied in tandem, TCC is a carbanilide (the two aromatic rings are linked by urea), whereas triclosan is a diphenyl ether (with oxygen linking the rings) [3]. Because of this, they may have different biological activity [3, 4].

Due to concerns regarding the effects of TCC on human health, exposure has been assessed in human populations residing in China, Europe, Canada, and the US. In the large-scale US biomonitoring study NHANES (2013-2014), TCC was detected in human urine in ~36% of individuals with concentrations up to 68 *μ*g/L. Sex (male), age (20+ years), race (non-Hispanic black), and increased body surface area were significantly associated with higher concentrations of TCC [5]. In another US study of pregnant women in Brooklyn, TCC had a detection frequency of 86.7% and its metabolites 2′-OH-TCC, 3′-OH-TCC, and 3′-Cl-TCC were found in 27.1%, 16.6%, and 12.7%, respectively [6]. In a study done by the US Centers for Disease Control (CDC), TCC was detected in 35% of urine samples tested, and the metabolites 2′-OH-TCC and 3′-OH-TCC were detected in 16% and 14.5% of urine samples, respectively. In the same study, serum TCC was detected in 44% of samples (although the metabolites were not detected above the limit of detection (LOD)). TCC was again detected more frequently in males than in females in the CDC study [7].

Interestingly, a Canadian biomonitoring study (the Canadian Health Measures Survey [CHMS]) found that only 4% of the population had detectable urinary TCC [8]. In another Canadian study, TCC was detected in less than 4% of infant urine and meconium samples and was not detected in infant formula or breast milk [9]. The authors attributed their findings, corroborated by the more recent study, to limited use of the chemical in Canada [9]. Similarly, a study done in Greece found a detection rate of 4% in urine with a mean level of 0.6 ng/mL (LDQ-1.9 ng/mL) [10]. In a 2013 Danish study done in mother-child pairs, TCC was detected at levels over the LOD in 25% of mothers and 28% of children, with mean TCC levels of 0.08 (LOD-1.3) and 0.07 (LOD-1.0) [11]. In a 2014 study of Danish pregnant women, TCC was found in 54% of samples tested [12]. When measured in children/adolescents, young men, and pregnant women, the compound was detected in 54%, 17%, and 18% of samples, respectively [13]. A study in a Chinese population found TCC in almost all (99-100%) of the urine samples tested, with high concentrations detected in the toenails and fingernails of the subjects [14]. Clearly, exposure to TCC varies by populations, with location, sex, age, and race/ethnicity possibly playing a role. This is likely due to the variable use of the chemical in consumer products by countries and individuals.

There has been very little research on the indirect exposure of offspring during development. However, in two studies by Enright et al., TCC has been shown to transfer to the fetus via maternal exposure in both human placental* in vitro* and rodent* in vivo* models [15, 16]. In the* in vivo* models, TCC was found in maternal and fetal placental tissue, as well as in the fetus. TCC also transfers to offspring during lactation in exposed mothers [15, 16].

TCC is an environmental contaminant (it was one of the most commonly detected organic wastewater and surface water contaminants in the US in 2011), with concentrations as high as 6.75 *μ*g/L. It is toxic to aquatic organisms (perhaps more so than triclosan), with an LC50 (i.e., the concentration of 50% lethality) as low as 3.1 *μ*g/L in certain organisms [17]. It has been shown to bioaccumulate in aquatic organisms, such as snails [17, 18]. In fish, TCC also bioaccumulates. However, TCC has been shown to be rapidly metabolized and eliminated after absorption in fish, facilitated by the* in vivo* conversion of TCC to its sulfate and glucuronic acid conjugate metabolites (i.e., 2′-O-Gluc-TCC, 2′-O-SO3-TCC, 2′-OH-TCC, 3′-OH-TCC, and 6-OH-TCC). Thus, the bioaccumulation in fish is less than would be predicted by the n-octanol/water partition coefficient of TCC [19].

The route of metabolism and the metabolites produced are similar in humans and other mammalian species [20–24]. In humans exposed orally to TCC, the urinary elimination half-life was about 20 hours [21]. With intravenous injection, the urinary elimination half-life was estimated to be 10 hours. In the same study, individuals were exposed to TCC through a whole body wash in the shower. The urinary elimination half-life in this case was 28 hours; however, the bulk of excretion was detected in the feces, which was detected 12–20 days after exposure [25]. The fact that some human populations had up to 100% detection indicates the ubiquitous exposure of TCC in certain groups [14].

Although TCC has been widely used for over 50 years, it was only recently that concerns were raised about its endocrine disruptive properties. In 2016, the FDA banned its use in over-the-counter hand and body washes [26]. This was due to the fact that manufactures were unable to show that TCC was safe for daily use or was any more effective than regular soap alone. However, TCC is still used in other countries (as evidenced by human exposure) [11, 12, 14] and the chemical is still approved in the US for all other personal care products, including deodorants, lotions, and toothpaste, as well as for medical and healthcare applications [26]. A 2007 study put forth the idea that TCC was a “new kind of endocrine disruptor” that augmented the action of endogenous hormones rather than directly activating hormone receptors [27]. More recently, TCC has been shown to disrupt the gut microbiome in animals [28] and humans [29], which, in turn, can have myriad effects on health [30]. Because of these potential endocrine-disruptive properties of TCC and the potential for TCC to be transferred to offspring during development, we conducted a scoping review to catalogue and map the literature-to-date on the endocrine, reproductive, and developmental effects of TCC.

## 2. Methods

### 2.1. Literature Search and Study Identification

A comprehensive literature search was performed to identify studies describing the endocrine, reproductive, and developmental effects of exposure to TCC. The search included all articles published for all years to August 2017. Electronic searches were performed in Web of Science, PubMed, and TOXLINE using the following search criteria: triclocarban OR trichlocarban OR triclocarbanum OR trilocarban OR (N-(4-chlorophenyl-N-(3,4-dichlorophenyl)) urea) OR (N-(3,4-dichlorophenyl-N-(4-chlorophenyl) urea) OR (1- (3′,4′-Dichlorophenyl) -3- (4′-chlorophenyl)) urea) OR trichlorocarbanilide OR trichlorodiphenylurea OR 101-20-2. 101-20-2 is the Chemical Abstracts Services (CAS) Registry Number for triclocarban. For inclusion, the studies had to be original works, be in the English language, and assess endocrine, reproductive, or developmental effects of triclocarban exposure, including human,* in vitro*, and* in vivo* models. All titles and abstracts were screened for inclusion independently by two reviewers using the software DistillerSR® (Evidence Partners, Ottawa, Ontario, Canada). Conflicts or discrepancies were resolved by the two reviewers.

### 2.2. Data Extraction

Parameters collected during data extraction included author information, year of publication, summary of endpoint(s) evaluated, information about the model used (e.g., cell line and species), concentrations/doses tested, exposure duration, age at exposure, and route of exposure. The data extraction entries were inputted by one reviewer and then quality-checked by a second reviewer. Any discrepancies were resolved by discussion.

## 3. Results

Our searches for potentially relevant studies resulted in 1560 articles. A total of 32 studies (2 human, 19* in vivo* animal, and 11* in vitro* only) were identified as relevant and these studies underwent full review and data extraction. The summary statistics of the body of literature are presented in Table 1. The majority of the research on this topic has been published in the years since 2010, with over 90% of the literature published in the past 7 years. Studies were identified in all three categories: development, endocrine, and reproduction. Doses in aquatic studies ranged from 100 ng/L to 10000 *μ*g/L, with many studies in the range of the 6.75 *μ*g/L found in US surface waters. The mammalian studies generally exposed animals to high doses, although a recent 2017 study used 100 nM, with the authors indicating that this was an “environmentally relevant concentration” [16].

### 3.1. Development

There were 11 studies identified with developmental endpoints, including two human studies. The human studies are presented in Table 2 and the animal studies in Table 3. The human studies assessed fetal malformations [31] and other birth parameters (gestational age at birth, birth weight, and body length/head size) [32]. Two studies in rodents examined birth and offspring weight [16, 33]. Other studies in rotifers [34], crustaceans [35, 36], echinoderms [37], mollusks [38], and fish [37, 39, 40] measured endpoints in offspring such as lifespan/survival, size, malformations, and altered gene expression.

### 3.2. Reproduction/Endocrine

Thirteen studies assessed the reproductive/endocrine effects of TCC* in vivo *(Table 3). Four studies assessed effects in rodents including sex organ weights (males) [27, 41], reproduction and mammary gland disruption (females) [33], and activity of the constitutive androstane receptor (CAR) [42]. One of these studies assessed steroidal augmentation of TCC [27], while the other three looked at the effects of TCC alone. Other species studied were a crustacean [43], mollusks [44, 45], and fish [39–41, 46–49]. Endpoints included fecundity, spermatogenesis/oogenesis, endogenous steroid levels, masculinization, and steroid-induced gene/protein expression (including vitellogenin).

### 3.3. *In Vitro*/Steroid Activity

In systematic review frameworks, such as the Office of Health Assessment and Translation (OHAT) Systematic Review Framework,* in vitro* mechanistic evidence can be quantitatively used to support health hazard conclusions [50]. The* in vitro* studies are presented in Table 4. Fourteen studies assessed the steroidal activity of TCC, which may support the biological plausibility of the reproductive/endocrine effects of TCC* in vivo *[27, 41, 42, 51–61]. These studies measured steroid and steroid receptor activity, including estrogen, androgen, progesterone, thyroid, and glucocorticoid, as well as CAR activity.  Table 5 shows the studies organized by the specific steroidal activity assessed. Some of these studies tested the possible steroid-enhancing activity of TCC, while others looked at the direct activities of TCC alone.

## 4. Discussion

Scoping reviews systematically map areas of research and can have several applications. They are employed during the first step of a systematic review in order to identify sources and types of available research to determine the feasibility/utility of carrying out a systematic review and to prioritize endpoints that can be evaluated through systematic review. They can also be used to help identify data gaps and direct future research. They have been used in the clinical sciences for many years [62–64] and have more recently been employed as tools in environmental health [65, 66]. While scoping reviews are extremely useful for generating hypotheses for systematic review, they do not themselves answer research questions (e.g., whether or not a chemical is harmful to human or environmental health) [67–70]. In this scoping review, we aimed to identify and map the literature-to-date of the endocrine, developmental, and reproductive effects of TCC on organisms in order to identify endpoints for future systematic review and determine if additional research is needed in specific areas.

### 4.1. Possible Systematic Review Endpoints

Our recommendations for future systematic reviews are based on several variables. In particular, endpoints suitable for systematic review ideally have literature in all three “evidence streams” (human, animal, and* in vitro*), although literature in all streams is not required for final analyses. Meta-analysis, which usually requires at least three studies on a specific endpoint, is an optional component of systematic reviews, and is especially useful when there are “conflicting” results from studies. Meta-analyses are helpful for determining the degree of hazard, if any, which is posed to humans [50]. The characteristics of the recommended systematic review endpoints are presented in Table 6.

When examining reproductive/endocrine endpoints, there is sufficient literature on the estrogenic and/or androgenic effects of TCC in animals to undertake a systematic review, including three rodent studies and eight nonmammalian studies. Further, there are many* in vitro* studies that might support the biological plausibility of the estrogenic/androgenic action of TCC, as most of the* in vitro* studies assessed these endpoints (Table 5). Although there are no human studies assessing these endpoints, there are sufficient studies (5) to evaluate vitellogenin expression in a meta-analysis, if desired. Vitellogenin is an estrogen-responsive egg protein in egg-laying animals and, when found in males, indicates exposure to estrogenic chemicals [71]. Both the animal and* in vitro* literatures in this area have studies examining the direct effects of TCC as well as steroid augmentation effects. A review employing systematic methodologies, comparing these two types of effects, could shed light on the mechanisms of TCC activity.

There are fewer studies on specific developmental endpoints, and therefore the developmental effects of TCC may not yet be mature enough for conducting a systematic review. One possible endpoint to pursue, however, is offspring size (birthweight and lifetime weight). This endpoint is represented in 1 human study [32] and 6 animal studies (2 rodent and 4 nonmammalian) [16, 33, 35, 37, 39, 40]. The fact that all three data streams are represented is a positive aspect for systematic review. There is also mechanistic evidence that could possibly be used to support the biological plausibility of TCC's effects on offspring growth, for example, TCC's effects on metabolic genes* in vivo* [16] and TCC activity on the thyroid receptor* in vitro* [55]. In all, however, there is little research on specific developmental endpoints.

### 4.2. Future Research

In order to protect human and wildlife health as quickly and efficiently as possible, further research of the effects of TCC should be prioritized. Overall, studies designed to be relevant to human health and risk assessment are needed, including identifying sensitive target tissues and functional assays, employing doses within the range of human exposure and using relevant models.  Table 7 outlines the state of the current research and identifies data gaps with respect to model and endpoint. While mechanistic models seem to be well represented, human and mammalian models are less so.

There are also data gaps with respect to endpoint. One endpoint that appears to be particularly responsive to low exposures (i.e., comparable to those reported in humans) is increased weight in offspring following developmental exposure [16]. This should be further explored in developmental animal studies and prospective epidemiological studies that follow the growth of children.* In vitro* studies exploring possible mechanisms for these actions would add to the body of literature as well. In general, more human studies would be helpful in understanding the effects of TCC, as we only found two human studies documenting the effects of TCC exposure.

For endocrine/reproductive effects, the majority of the literature is in aquatic species. Endpoints in these papers, such as vitellogenin induction, appear to be sensitive to low doses of TCC. However, the current endocrine/reproductive literature in mammals is less robust, and the endpoints studied, such as reproductive organ weights, are not as sensitive. Further research in this area should include mammalian models, with endpoints that are responsive to low doses of chemicals, such as ovarian histology and mammary disruption.

Lastly, the finding that TCC acts as a “steroid augmenter” should be studied further. The ability of endocrine disruptors to enhance the activity of endogenous hormones has not been extensively explored. TCC (and triclosan) appears to act this way* in vivo* and* in vitro*, and this may be a unique characteristic of these chemicals. Further study of this mechanism (and inclusion of assays that identify this activity among more chemicals) could be informative to the broader field of endocrine disruption.

## 5. Conclusions

Our scoping review found that systematic review(s) of the effects of TCC on human health could be carried out using the current literature, using a framework such as the OHAT Systematic Review Framework [50] or the Navigation Guide [69, 70]. The best candidate endpoints are the estrogenic and/or androgenic effects and offspring birth weight/growth. However, future research could add to the body of literature of each of these endpoints and improve the confidence of the results of any systematic reviews carried out. In particular, there are very few mammalian studies and almost no human studies. Thus, future research should include animal and human developmental growth studies and mammalian studies that include sensitive endocrine/reproductive endpoints. Systematic reviews of these topics, especially with the goal of deriving human health hazard conclusions of TCC effects, could aid in future research and regulation in order to most effectively protect human health.

## Figures and Tables

**Table 1:** Summary of the studies evaluating the endocrine, reproductive, and developmental effects of triclocarban.

	Number of studies (%)
*Total number of studies*	32
*Publication date*	
2010–2017	29 (91)
2000–2009	2 (6)
1969–1999	1 (3)
*In vivo studies*	18 (56)
Human	2 (6)
Animal	13 (41)
Mouse	2 (6)
Rat	3 (9)
Fish	7 (22)
Mollusk	3 (9)
Crustacean	3 (9)
Echinoderm	1 (3)
Rotifer	1 (3)
*In vitro studies*	14 (44)
*Age of exposure*	
Embryonic	3 (9)
Neonatal	3 (9)
Larval	3 (9)
Juvenile	3 (9)
Adult	10 (31)

**Table 2:** Human studies evaluating the developmental effects of triclocarban.

Author	Number of subjects	Age of subjects at time of exposure	Concentration detected	Exposure measurement	Summary
Geer et al., 2017	185	Adult; prenatal	0.13 *µ*g/L (cbp); 3.44 *µ*g/g creatinine (urine)	Maternal urine and cord blood	Cord blood plasma TCC was associated with decreased gestational age at birth in an immigrant population in Brooklyn, New York, in linear models adjusted for demographic confounders. The TCC metabolite 3′-Cl-TCC in third-trimester maternal urine was associated with fewer low birth weights.

Wei et al., 2017	92	Adult; prenatal	0.248 ng/mL (ms); 0.105 ng/mL (cs)	Maternal venous and umbilical cord blood	TCC levels were determined in a case-control study of mothers with abnormal births set in Beijing, China. Cases had one or more fetal anomalies and controls had no malformations. Levels of TCC in maternal sera were correlated with levels in cord blood. There was no difference in TCC in maternal sera or cord blood between cases and controls.

ms: maternal serum; cs: cord serum; cbp: cord blood plasma.

**Table 3:** Animal studies evaluating the endocrine/reproductive and developmental effects of triclocarban.

Study	Model	Strain	Exposure duration	Age at exposure	Route of exposure	Doses	LOEL	Summary
Reproductive/endocrine								

Ankley et al., 2010	Fish	Fathead minnow *(Pimephales promelas)*	21 d	Adult	Submersion	5, 10 *µ*g/L	5.00 *µ*g/L	TCC alone did not masculinize females (measured by induction of tubercles), but TCC + trenbolone increased tubercle scores, enhancing the effects of TRB alone. TCC had no effect on VTG levels.

Barros et al., 2017	Crustacean	*Gammarus locusta*	60 d	Juvenile, adult	Submersion	100, 500, 2500 ng/L	NA	TCC disrupted biochemical responses in *Gammarus locusta* exposed to environmentally relevant levels for 60 d but had no effect on ecological responses (survival, body length, or reproduction).

Chen et al., 2008	Rat	Sprague Dawley	10 d	Adult	Oral (food)	0.25%	NA	Rats exposed to T + TCC showed increased weights of seminal vesicles, ventral prostate, glans penis, Cowper's gland, and LABC muscle compared to T treatment alone. TCC treatment alone increased ventral prostate weight.

Chung et al., 2011	Fish	Zebrafish *(Danio rerio)*	24 h	Embryonic	Submersion	0.25 *µ*M	NA	TCC alone did not induce AroB expression (which is estrogen-responsive), but it enhanced E2-induced AroB expression. TCC also inhibited BPA-induced AroB expression.

Duleba et al., 2011	Rat	Sprague Dawley	10 d	Adult	Oral (food)	0.25%	NA	TCC treatment induced changes in the wet weight of the liver, seminal vesicle, ventral prostate, LABC, and glans penis. The dry weight of the seminal vesicle, LABC, and glans penis was increased by TCC exposure. TCC also increased the protein and DNA content of the ventral prostate, LABC, and glans penis. LH and T levels were not affected.

Geiss et al., 2016	Mollusk	Mud snail* (Potamopyrgus antipodarum)*	28 d	Embryonic	Submersion	0.1, 0.3, 1, 3, 10 *µ*g/L (NC)	0.3 *µ*g/L	Chronic exposure to environmentally relevant levels of TCC for 28 days altered the number of embryos in the brood pouch of mud snails in a nonmonotonic fashion.

Giudice et al., 2010	Mollusk	Mud snail* (Potamopyrgus antipodarum)*	2, 4 w	Adult	Submersion	0, 0.045, 0.14, 0.45, 1.4, 4.5, 14.0 *µ*g/L	0.14 *µ*g/L	After 4 weeks (but not before), TCC-exposed snails had significantly increased unshelled, shelled, and total embryos (there were some nonmonotonic results with shelled and total embryos).

Kennedy et al., 2014	Rat	Sprague Dawley	35 d	Embryonic, adult	Oral (food)	0.2, 0.5% w/w TCC	0.2% w/w TCC	TCC decreased maternal body weight gain and circulating T3 during gestation. There was no effect on implantation number, maternal organ weights, or hormone profile (E2, P, T, T4, and TSH). Exposure to pups from gestation through lactation did not affect number of pups born or birth weight. However, pups exposed to TCC from gestation through lactation did not survive past PND8 and there was evidence of mammary gland involution in the dams. Body weight and survival were decreased in pups nursed by TCC exposed dams.

Schultz et al., 2012	Fish	Fathead minnow *(Pimephales promelas)*	12 d	Larval, adult	Submersion	550, 1600 ng/L	1600 ng/L	In adults, TCC exposure had no effect on GSI, SSC, gonad histology, or VTG.

Villeneuve et al., 2017	Fish	Fathead minnow *(Pimephales promelas)*	22 d	Adult	Submersion	1, 5 *µ*g/L	1 *µ*g/L	Fecundity was decreased by greater than 50% in fathead minnows exposed to 5 *µ*g/L TCC but not in those exposed to 1 *µ*g/L. Fecundity was further decreased with coexposure to 5 *µ*g/L TCC and 0.5 *µ*g/L 17b-trenbolone. Chronic exposure to TCC for 22 d did not affect GSI or male secondary sex characteristics and did not cause masculinization of female fish. This exposure also did not alter plasma VTG concentrations or circulating E2 or T concentrations. In contrast, *ex vivo* assessment of steroid production from the reproductive organs was altered by TCC. Fish exposed to 5 *µ*g/L TCC had more preovulatory atretic follicles and other abnormalities. Gene expression changes in the ovary were also observed in fish exposed to 5 *µ*g/L TCC.

Wang et al., 2016	Fish	Zebrafish *(Danio rerio)*	21 d	Adult	Submersion	2.5, 5 *µ*g/L (NC)	2.5 *µ*g/L	The effects on reproduction of TCC alone or in combination with mercury were examined following 21 days of exposure. TCC exposure reduced spermatogenesis in males and delayed maturation of oocytes in females. Serum T and E2 were decreased in fish of both sexes and the expression of 3*β*-HSD, CYP17, 17-*β*-HSD, and CYP19a was decreased in the testes and disrupted in the ovaries of exposed fish. Liver VTG expression was also altered in males.

Yueh et al., 2012	Mouse	hUGT1^*∗*^28 and CAR-null	2 d	Adult	Intraperitoneal	16, 20 mg/kg	16 mg/kg	TCC exposure in transgenic mice resulted in increased hUGT and CYP gene expression via the CAR.

Zenobio et al., 2014	Fish	Fathead minnow *(Pimephales promelas)*	48 h	Adult	Submersion	1.40 *µ*g/L	NA	TCC exposure did not impact mortality, condition factor, or GSIs in adults. TCC exposure resulted in increased expression of liver VTG in both males and females, decreased testis AR and StAR, increased liver LPL in males, and decreased ovarian AR expression. There were no changes in CYP19a, ER*α*, THR*α*, or PGES.

Developmental								

Davis and Hidu, 1969	Mollusk	Clams and oysters	10 d and 12 d	Larval	Submersion	.0025, .005, .01, .025, .05, .1, .25, .50, 1.00 ppm	.01 ppm	TCC caused complete lethality of developing clam eggs larvae at 0.05 ppm and 0.1 ppm, respectively.

Enright et al., 2017	Mouse	CD-1	GD1–18; PND0–10	Embryonic; neonate; adult	Oral (water)	100 nM	NA	TCC-exposed offspring had increased bodyweight compared to controls, which persisted to PND56 (after cessation of treatment at PND10). Brain (both sexes) and uterine weights (females) were reduced in offspring. Fat pad and thymus weights were increased in female offspring. Also, in females, gene expressions of several lipid metabolism genes including leptin, adiponectin, and PPAR*α* were downregulated in adipose and liver tissues.

Han et al., 2016	Rotifer	*Brachionus koreanus*	3, 6, 12, 24 h and 1–10 d	Neonate	Submersion	50, 100, 200 *µ*g/L	100 *µ*g/L	TCC retarded population growth and reduced cumulative offspring and lifespan of *Brachionus koreanus*. TCC also altered the expression of xenobiotic metabolizing genes.

Kennedy et al., 2014	Rat	Sprague Dawley	35 d	Embryonic, adult	Oral (food)	0.2, 0.5% w/w TCC	0.2% w/w TCC	TCC exposure to pups from gestation through lactation did not affect number of pups born or birth weight. However, pups exposed to TCC from gestation through lactation did not survive past PND8. Body weight and survival were decreased in pups nursed by TCC-exposed dams.

Schultz et al., 2012	Fish	Fathead minnow *(Pimephales promelas)*	12 d	Adult; larval	Submersion	550, 1600 ng/L	1600 ng/L	In the larva, TCC treatment did not have any effect on body weight, time to response, escape velocity, or total escape response.

Simon et al., 2015	Crustacean	*Daphnia magna*	14, 54, 68 d	Adult; juvenile; neonate	Submersion	EC 50: 13 *µ*g/L neonates 26 *µ*g/L adults 33 *µ*g/L juveniles	13 *µ*g/L	On a population level, TCC reduced population density of daphnia immediately following exposure. Mortality was size- and age-dependent with neonates being more sensitive than adults. Mortality was decreased when multiwalled carbon nanotubules were present in the growth medium. Exposure to TCC or TCC + multiwalled carbon nanotubules increased the ratio of juveniles to adults in the population but had no effect on minimal or maximal body length.

Torres et al., 2016	Fish; echinoderm	Zebrafish *(Danio rerio)*; sea urchin *(Paracentrotus lividus)*	8, 32, 48, 80 h	Embryonic	Submersion	0.1024, 0.256, 0.64, 1.6, 4, 10, 100, 350, 600, 850, 1000, 10000 *µ*g/L	0.64 *µ*g/L	TCC and other chemicals were tested in two embryo bioassays. TCC increased mortality rates at exposures greater than 350 *µ*g/L but did not have effects on other developmental parameters. The NOEC for zebrafish was 100 *µ*g/L. In *Paracentrotus lividus*, TCC decreased larval length and increased abnormalities at 0.64 and 1.6 *µ*g/L, respectively.

Villeneuve et al., 2017	Fish	Fathead minnow *(Pimephales promelas)*	22 d	Adult	Submersion	1, 5 *µ*g/L	1 *µ*g/L	Chronic exposure to TCC for 22 days did not affect adult body mass.

Xu et al., 2015	Crustacean	*Artemia salina*	6, 12, 24 h	Larval	Submersion	18.0 *µ*g/L	NA	TCC caused dose-dependent mortality in *Artemia salina* and was an order of magnitude more potent than TCS. DNA damage and apoptosis of *Artemia salina* nauplii coelomocytes were apparent as early as 12 and 24 hours after exposure

NC: nominal concentration; NOEC: no observable effect level; TCC: triclocarban; AroB: CYP19a1; BPA: bisphenol A; BCF: [(body weight/total length^3^) × 100000]; GSI: (gonad weight/whole-body × 100); SSC: secondary sex characteristic; hUGT: humanized uridine 5′-diphospho-glucuronosyltransferase; CAR: constitutive active/androstane; LABC: levator anibulbocavernosus; LH: luteinizing hormone; CYP19a: aromatase; ER*α*: estrogen receptor *α*; AR: androgen receptor; THR*α*: thyroid hormone receptor *α*; PGES: prostaglandin endoperoxide synthase; StAR: steroidogenic acute regulatory protein; dmrt: doublesex- and mab-3-related transcription factor; VTG: vitellogenin; LPL: lipoprotein lipase; AChE: acetylcholinesterase; T3: triiodothyronine; E2: estradiol; P: progesterone; T: testosterone; T4: thyroxine; TSH: thyroid-stimulating hormones; PND: postnatal day.

**Table 4:** Studies evaluating the *in vitro* activity of triclocarban.

Study	Model	LOEL	Concentrations tested	Summary
Ahn et al., 2008	BG1-ERE; H4L1.1c4-DRE; T47D-ARE; mMyoblasts	1 × 10^−7^ M	1 × 10^−9^, 1 × 10^−8^, 1 × 10^−7^, 1 × 10^−6^ M	TCC enhances the ER- and AR-mediated activity of E2 and T, respectively.

Blake et al., 2010	MDA-kb2	1000 nM	Cells dosed from 125 to 2,000 nM	TCC induced AR-activated luciferase activity alone and in conjunction with 17*β*-trenbolone. Activity was significantly higher than control at a range from 1000 to 2000 nM TCC.

Chen et al., 2007	2933Y; JK293; MDA-kb2	0.5 *µ*M	0.1, 0.5, 1.0 *µ*M	TCC did not induce cell proliferation in the MTT assay. TCC enhanced T-mediated AR activity over T-treatment alone.

Christen et al., 2010	MDA-kb2	NA	0.05, 0.5, 5 *µ*M	TCC showed no *in vitro* androgenic activity.

Duleba et al., 2011	C4-2B; LNCaP	1.0 *µ*Mol/L	1.0 *µ*Mol/L	TCC induced androgen receptor activity only when administered with DHT or T.

Gao et al., 2015	*Tetrahymena thermophila*	1 *µ*g/L	1, 10, 100, 250, 500, 750, 1000, 2000, 4000 *µ*g/L	TCC inhibited the growth of *Tetrahymena thermophile* and was more potent than TCS. At environmentally relevant levels, TCC caused DNA damage and at higher levels TCC impaired the plasma membrane resulting in decreased cell viability. TCC inhibited efflux transporter activities by downregulating the expression of the membrane efflux protein Abcb15.

Hinther et al., 2011	GH3	10 nM	10, 100, 1000 nM	In the C-fin assay, TCC reduced *Rana* larval keratin I (RLKI, a TH-repressed gene) but not TR*β* expression (a TH-induced gene). T3 + TCC exposure showed no differences in these genes. TCC also induced HSP30 and CAT expression (measures of cellular stress response). T3 + TCC exposure induced HSP30 but not CAT. In the GH3 assay, TCC alone decreased GH, Dio1, PRL, and HSP70 expression, but TCC + T3 increased GH, Dio1, and HSP70; PRL levels were still decreased (nonmonotonically).

Huang et al., 2014	CV-1; MCF-7	1 × 10^−7^ M	1 × 10^−9^, 5 × 10^−9^, 1 × 10^−8^, 5 × 10^−8^, 1 × 10^−7^, 5 × 10^−7^, 1 × 10^−6^ M	TCC was shown to be a weak estrogen agonist. TCC induced a dose-dependent response in the ER*α* reporter gene assay and MCF7 proliferation assay (E-Screen). Cell proliferation was mediated by ER*α* as cotreatment with ICI 182,780 blocked cell growth. Protein and mRNA expression of pS2 were increased, while those of ER*α* were decreased. The expression of several miRNAs (miR-22, -206, and -193b) that regulate ER-*α* was upregulated with TCC exposure.

Kolšek et al., 2014	MDA-kb2	2 *µ*M	2 *µ*M	TCC produced amplification of the T and GR response in the assays assessing antagonism by an unknown mechanism. TCC showed cytotoxicity at concentrations higher than 2 *µ*M. TCC did not appear to be an AR or GR agonist or antagonist in reporter gene assays.

Simon et al., 2014	H295R; RTL-W1; T47D	125 *µ*g/L	31.25, 62.50, 125, 250, 500 *µ*g/L; ~0.01–10 mg/L	Antiestrogenic activity of TCC was shown at 125 *µ*g/L and higher. The addition of CNT ameliorated the effects of TCC. Production of E2 was not impacted by TCC exposure in H295R cells and the compound did not induce ROS in any of the tested cell lines.

Tarnow et al., 2013	HeLa9903; MCF-7; MDA-kb2	1 *µ*M		TCC enhanced estrogen and androgen activity mediated through ER and AR, respectively. TCC did not enhance the expression of DHT-induced AR target genes. TCC did not enhance E2-induced proliferation in the E-Screen assay. TCC enhanced estrogen (E2, butyl paraben, BPA, genistein)-induced expression of CYP1A1 and CYP1B1 in MCF-7 cells, mediated through the AhR pathway.

Tonoli et al., 2015	H295R	0.5 *µ*M	.5, 1, 2.5, 10 *µ*M	TCC exposure caused altered adrenal steroidogenesis by affecting an early step in steroid biosynthesis. Pregnenolone, progesterone, 11-DOC, 17*α*-hydroxyprogesterone, and DHEA were the most sensitive to increasing TCC concentrations. 11-Dehydrocorticosterone, androstenedione, and DHEAS were markedly decreased and aldosterone and cortisone were slightly decreased.

Wu et al., 2016	FRTL-5	0.3 *µ*M	0.03, 0.1, 0.3, 1, 3, 10, 30, 100, 300 *µ*M; 20, 40, 80 *µ*M	TCC disrupts thyroid hormone homeostasis. TCC decreased NIS-mediated iodide uptake in a noncompetitive manner. The expression of three genes involved in TH synthesis (Slc5a5, TPO, and Tg) or thyroid transcription factors (Pax8, FoxE1, and Nkx2-1) was not altered by TCC exposure. TCC was a weak inhibitor of TPO activity, indicating that TPO may not be a primary target of TCC.

Yueh et al., 2012	CV-1; MCF-7; MDA-MB-231	1 *µ*M	10 *µ*M	TCC activated ER*α* and CAR in vitro, in luciferase reporter gene transfected cell lines, but not ER*β*, PXR, LXR, FXR, PPARs, and GR. TCC induced expression of CYP genes in cells that contained ER*α* (MCF-7) but not in MDA-MB-231 cells which do not contain ER*α*.

TCC: triclocarban; ER: estrogen receptor; AR: androgen receptor; E2: 17*β*-estradiol; T: testosterone; DHT: dihydrotestosterone; BPA: bisphenol-a; AhR: aryl hydrocarbon receptor; TR*β*: thyroid receptor *β*; HSP30: heat-shock protein 30; CAT: catalase; GH: growth hormone; PRL: prolactin; HSP70: heat-shock protein 70; CAR: constitutive androstane receptor; PXR: pregnane X receptor; LXR: liver X receptor; PPAR: peroxisome proliferator-activated receptor; GR: glucocorticoid receptor; MTT: 3-(4,5-dimethylthiazol-2-yl)-2,5-diphenyltetrazolium bromide; 11-DOC: 11-deoxycorticosterone; DHEA: dehydroepiandrosterone; DHEAS: dehydroepiandrosterone sulfate; NIS: sodium-iodide symporter; Slc5a5: sodium-iodide symporter gene; TPO: thyroperoxidase; Tg: thyroglobulin; Pax8: paired box gene 8; FoxE1: thyroid transcription factor 2; Nkx2-1: thyroid transcription factor 1; Abcb15: ABC transporter B family member 15; CNT: carbon nanotubes; ROS: reactive oxygen species.

**Table 5:** Types of hormone systems assessed *in vitro.*

Study	E	A	T	G	P
Ahn et al., 2008	✓	✓			
Blake et al., 2010		✓			
Chen et al., 2007		✓			
Christen et al., 2010		✓			
Duleba et al., 2011		✓			
Gao et al., 2015					
Hinther et al., 2011			✓		
Huang et al., 2014	✓				
Kolšek et al., 2014		✓		✓	
Simon et al., 2014	✓				
Tarnow et al., 2013	✓	✓			
Tonoli et al., 2015				✓	✓
Wu et al., 2016			✓		
Yueh et al., 2012	✓			✓	

E: estrogenic; A: androgenic; T: thyroidogenic; G: glucocorticodogenic; P: progestrogenic.

**Table 6:** Description of recommended systematic review endpoints.

Number of studies	Endpoint		
	Estrogenic activity	Androgenic activity	Offspring growth
Human	0	0	1
Rodent	1	2	2
Fish	6	5	3
Invertebrates	2	0	1
*In vitro*	5	7	2^*∗∗*^

Total^*∗*^	14	14	9

Dose range			
Human (detected)	NA	NA	0.13 *µ*g/L (cord blood plasma); 3.44 *µ*g/g creatinine (urine)
Rodent	0.2–0.5% w/w	0.25% w/w	100 mM, 0.2–0.5% w/w TCC
Aquatic	0.045–14.0 *µ*g/L	0.55–10 *µ*g/L	0.1–10000 *µ*g/L
*In vitro*	1 × 10^−9^–1 × 10^−3^ M	1 × 10^−9^–2 × 10^-3 ^M	3 × 10^−6^–3 × 10^−2^ M

^*∗*^ *In vitro*/*in vivo* assessments counted separately, even if included in the same publication. ^*∗∗*^Thyroid activity.

**Table 7:** Research gaps of the effects of TCC on reproductive, endocrine, and developmental endpoints.

	Reproductive	Endocrine	Developmental
Human	−	−	+
Animal: mammals	+	+	+
Animal: nonmammals	+	+++	++
*In vitro*	−	+++	+

−: no research available; +: some research (1-2 studies); ++: moderate research (3-4 studies); +++: most research (5+ studies).
